# A framework for the interpretation of heart rate variability applied to transcutaneous auricular vagus nerve stimulation and osteopathic manipulation

**DOI:** 10.14814/phy2.15981

**Published:** 2024-03-20

**Authors:** Adrienne Kania, Jumana Roufail, Joseph Prokop, Harald M. Stauss

**Affiliations:** ^1^ Department of Clinical Medicine Burrell College of Osteopathic Medicine Las Cruces New Mexico USA; ^2^ Department of Biomedical Sciences Burrell College of Osteopathic Medicine Las Cruces New Mexico USA

**Keywords:** cardiac parasympathetic tone, cardiac sympathetic tone, frequency‐domain heart rate variability, time‐domain heart rate variability

## Abstract

Reports on autonomic responses to transcutaneous auricular vagus nerve stimulation (taVNS) and osteopathic manipulative techniques have been equivocal, partly due to inconsistent interpretation of heart rate variability (HRV). We developed a mechanistic framework for the interpretation of HRV based on a model of sinus node automaticity that considers autonomic effects on Phase 3 repolarization and Phase 4 depolarization of the sinoatrial action potential. The model was applied to HRV parameters calculated from ECG recordings (healthy adult humans, both genders) before (30 min), during (15 min), and after (30 min) a time control intervention (rest, *n* = 23), taVNS (10 Hz, 300 μs, 1–2 mA, cymba concha, left ear, *n* = 12), or occipitoatlantal decompression (OA‐D, *n* = 14). The experimental protocol was repeated on 3 consecutive days. The model simulation revealed that low frequency (LF) HRV best predicts sympathetic tone when calculated from heart rate time series, while high frequency (HF) HRV best predicts parasympathetic tone when calculated from heart period time series. Applying our model to the HRV responses to taVNS and OA‐D, revealed that taVNS increases cardiac parasympathetic tone, while OA‐D elicits a mild decrease in cardiac sympathetic tone.

## 1 INTRODUCTION

Since the pioneering work by Akselrod et al. (1981), heart rate variability (HRV) analysis has been used extensively to assess cardiac autonomic responses to physiologic (Furlan et al., 2000), environmental (Bruce‐Low et al., 2006), and mental perturbations (Moriguchi et al., 1992), in disease states (Kleiger et al., 1987; Malpas & Maling, 1990), and in response to therapeutic interventions (Carandina et al., 2023; Heusser et al., 2003; Pelat et al., 2003). HRV analysis tools are now readily available and have become an integral part of EKG monitors and even cell phone applications (Moya‐Ramon et al., 2022). While easy access to such tools is desirable, it also comes with the risk of overly simplifying the interpretation of HRV data (Berntson et al., 1997; Pagani & Malliani, 2000). A commonly found interpretation of HRV is that the square root of the mean of the sum of the squares of differences between adjacent NN intervals (RMSSD) and the high frequency (HF) spectral power of RR‐interval variability are “markers for parasympathetic activity” and that low frequency (LF) spectral power of RR‐interval variability is a “marker for sympathetic and parasympathetic activity” (Curi et al., 2018; Forte et al., 2022; Giles et al., 2013; Machetanz et al., 2021). However, there has been a long ongoing debate as to if HRV‐derived parameters are indeed “markers for parasympathetic or sympathetic activity” (Parati et al., 2006; Taylor & Studinger, 2006).

In one of the early landmark studies on cardiovascular variability, Pagani et al. (1986) demonstrated that interventions, such as 90° tilt, nitroglycerin‐induced hypotension, and coronary artery occlusion that all activate the sympathetic nervous system, are associated with increased LF spectral power of RR‐interval variability. Furthermore, Pomeranz et al. (1985) demonstrated that parasympathetic blockade with atropine reduces LF and HF RR‐interval variability, while sympathetic blockade with propranolol selectively blocks LF RR‐interval variability. Furthermore, ganglionic receptor blockade with trimethaphan reduces LF and HF RR‐interval variability (Diedrich et al., 2002). These and similar studies are often referenced in support of the concept that HRV‐derived parameters are markers for autonomic activity. However, few investigators use a mechanistic framework for the interpretation of HRV parameters that is based on the effects of cardiac sympathetic and parasympathetic innervation on sinoatrial automaticity. Specifically, the effects of M_2_‐muscarinic and β_1_‐adrenergic receptors on the electrophysiologic properties of the spontaneous action potentials in cardiac sinus node cells need to be considered when linking HRV to cardiac autonomic activity.

Such an approach has been used by Monfredi et al. (2014) to investigate the relationship between heart rate variability and the mean level of heart rate. Using two biophysical models, these authors concluded that “heart rate variability is primarily dependent on heart rate and cannot be used in any simple way to assess autonomic nerve activity to the heart” (Monfredi et al., 2014). However, as pointed out in an editorial to the article by Monfredi et al. (Stauss, 2014) the dependency of heart rate variability from the mean level of heart rate can partly be overcome by calculating heart rate variability from heart rate time series instead of from heart period (RR‐interval) time series, which is the currently accepted standard (Task Force of the European Society of Cardiology and the North American Society of Pacing and Electrophysiology, 1996). Therefore, we used a biophysical model of sinus node automaticity similar to those described by Monfredi et al. (Monfredi et al., 2014) to build a framework for the interpretation of heart rate variability that is based on heart rate variability computed from heart rate and from heart period time series. It would be desirable to validate the biophysical model by experimental data. However, this would require recording the action potentials of the sinus node cells in vivo, which is currently impossible to achieve. The alternative approach to build a model around experimental data would require knowledge of the cardiac autonomic responses to experimental interventions, which is also impossible to directly obtain in vivo, because it would require assessing sympathetic and parasympathetic nerve activity directed to the sinus node cells. Thus, we used a biophysical model of sinus node automaticity and autonomic control of sinus node cells to build a framework for the interpretation of heart rate variability data and provide examples on how this framework may be applied to experimental data.

The objectives of this study were twofold: (1) to build a framework for the interpretation of heart rate variability data based on an established model of autonomic nervous system modulation of sinus node automaticity and (2) to apply this framework to the interpretation of HRV data obtained in response to noninvasive transcutaneous auricular vagus nerve stimulation (taVNS) and to the osteopathic occipitoatlantal decompression (OA‐D) technique. These two interventions are particularly interesting for the purpose of this study, because the interpretation of HRV responses to taVNS and to osteopathic manipulative treatment techniques has been debated (Carnevali et al., 2020; Cerritelli et al., 2020; Soltani et al., 2023; Wolf et al., 2021).

## 2 MATERIALS AND METHODS

### 2.1 Model of autonomic nervous system effects on variability of sinus node automaticity

**FIGURE 1:**
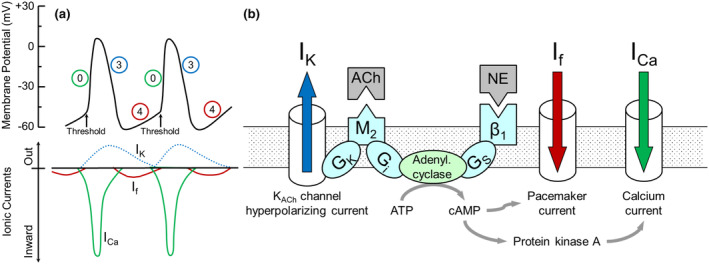
*Panel a (left)*: Action potentials in the sinoatrial node are initiated by a slow depolarization during Phase 4. Once a threshold level is reached, Phase 0 of the next action potential is initiated. The important ionic currents driving the action potentials in sinoatrial node cells are *I*_Ca_ (calcium current, Phase 0), *I*_K_ (potassium current, Phase 3), and *I*_f_ (funny current, Phase 4). *Panel b (right)*: Intracellular mechanisms of autonomic control of sinoatrial node function. Norepinephrine (NE) binds to ß_1_‐adrenergic receptors, which increases the activity of adenylyl cyclase via a stimulating G protein (G_S_). This raises intracellular cAMP, which increases the funny current *I*_f_ and the calcium current *I*_Ca_. Acetylcholine (ACh) binds to M_2_‐muscarinic receptors. This activates a G_K_ protein, which increases the open probability of acetylcholine‐dependent potassium channels (K_ACh_), leading to an increase in the hyperpolarizing potassium current *I*_K_. M_2_‐muscarinic receptors also inhibit adenylyl cyclase via an inhibitory G protein (*G*_i_) and, therefore, antagonize sympathetic, β_1_‐adrenergic receptor‐mediated effects on *I*_f_ and *I*_Ca_.

**FIGURE 2:**
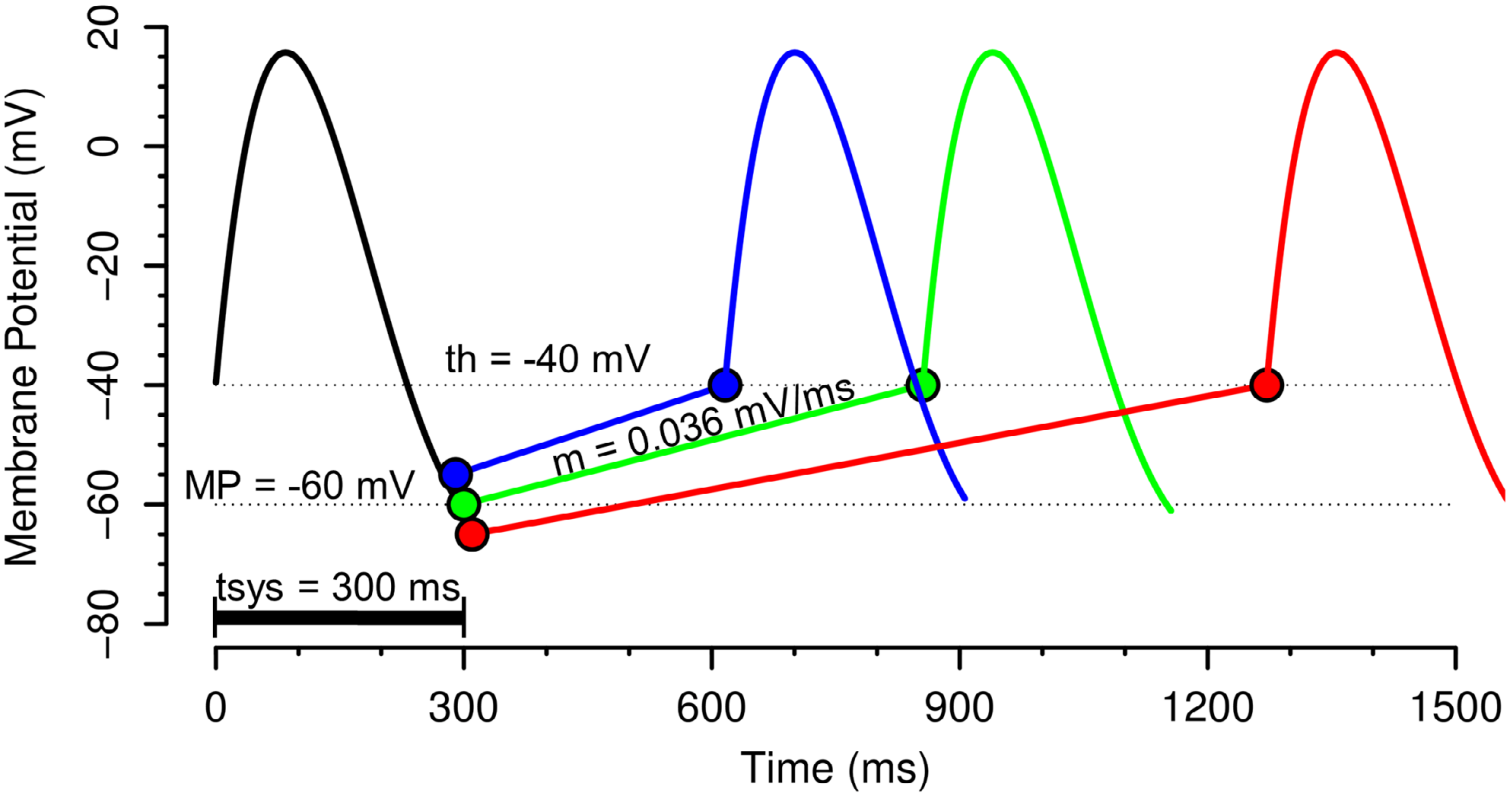
Autonomic modulation of action potentials in sinoatrial node cells. The duration from the beginning of Phase 0 to the end of Phase 3 is considered the time of systole (tsys). The model assumes that tsys is not affected by sympathetic (SNA) or parasympathetic nervous system activity (PNA). The membrane potential at the end of Phase 3 repolarization (MP) is affected by PNA through modulation of the potassium current *I*_K_ (see Figure 1b). SNA does not affect MP. The slope of Phase 4 depolarization (*m*) is affected by SNA and PNA through modulation of the activity of adenylyl cyclase and intracellular cAMP concentrations, affecting the funny current *I*_f_ (see Figure 1b). The model assumes that the threshold membrane potential for triggering a new action potential (th) is constant and not affected by SNA or PNA. The green line represents control conditions with tsys = 300 ms, MP = −60 mV, *m* = 0.036 mV/ms, and th = −40 mV. The blue line represents increased SNA combined with decreased PNA. In this example, MP is raised to −55 mV and m is increased to 0.046 mV/ms, resulting in a shortened heart period and increased heart rate. The red line represents increased PNA combined with decreased SNA. In this example, MP is lowered to −65 mV and m is reduced to 0.026 mV/ms, resulting in a lengthened heart period and decreased heart rate.

In developing a framework for the interpretation of HRV parameters, it is important to consider the electrophysiologic mechanisms driving sinoatrial node automaticity. As illustrated in Figure 1a, this automaticity largely depends on the slow diastolic depolarization during Phase 4 of the action potential that is driven by the funny current *I*_f_ that is now also known as the current through the hyperpolarization‐activated cyclic nucleotide‐gated (HCN) channels (*I*_H_). The automaticity also depends on the potassium current (*I*_K_) that drives repolarization during Phase 3, because it determines the starting membrane potential for the slow diastolic depolarization. In an isolated frog heart preparation, electrical stimulation of the vagus nerve resulted in an average increase in this potential (more negative) from −55 to −65 mV (Hutter & Trautwein, 1956). In the same study (Hutter & Trautwein, 1956), electrical stimulation of the vagosympathetic trunk did not alter the threshold potential required to initiate a new action potential. Thus, for our model, we assumed that autonomic nervous system function modulates Phase 3 repolarization (*I*_K_) and Phase 4 depolarization (*I*_f_) as illustrated in Figure 1b. The autonomic effect on the calcium current *I*_Ca_ would alter contractility of working cardiomyocytes, but appears to have little effect on sinoatrial automaticity. The parasympathetic nervous system through M_2_‐muscarinic receptors inhibits adenylyl cyclase, while the sympathetic nervous system through β_1_‐adrenergic receptors stimulates adenylyl cyclase (Figure 1b). Thus, fluctuations in cardiac autonomic tone result in corresponding fluctuations in intracellular cAMP, which modulate the funny current (*I*_f_) that drives Phase 4 depolarization. Cardiac parasympathetic innervation through M_2_‐muscarinic receptors also regulates *I*_K_ that determines Phase 3 repolarization. However, *I*_K_ is independent of sympathetic modulation. Importantly, fluctuation in intracellular cAMP concentrations can only occur slowly because they depend on enzymatic reactions involving cAMP synthesis by adenylyl cyclase and cAMP degradation by phosphodiesterase. These enzymatic reactions allow for fluctuations in intracellular cAMP that are consistent with the timing of LF HRV, but they are too sluggish to support HF HRV. Thus, HF HRV cannot be explained by cAMP‐dependent fluctuations in *I*_f_ driving Phase 4 depolarization. Instead, HF HRV depends exclusively on fluctuations in *I*_K_ (Phase 3 repolarization), which depend on parasympathetic M_2_‐muscarinic receptors, but not on sympathetic β_1_‐adrenergic receptors. Based on these considerations, our model of autonomic nervous system effects on variability of sinus node automaticity is based on the following assumptions as illustrated in Figure 2.

1. The time interval from the beginning of Phase 0 to the end of Phase 3 repolarization (tsys in Figure 2) is constant.
2. The threshold membrane potential for triggering a new action potential (th in Figure 2) is constant.
3. Fluctuations in heart period or heart rate are exclusively caused by fluctuations in the slope of Phase 4 depolarization (*m* in Figure 2, modulated by sympathetic and parasympathetic activity) and/or fluctuations in the membrane potential at the end of Phase 3 repolarization (MP in Figure 2, modulated only by parasympathetic activity).
4. Fluctuations in the slope of Phase 4 depolarization (*m* in Figure 2) are too sluggish to follow HF variability. Thus, HF variability exclusively depends on fluctuations in the membrane potential at the end of Phase 3 repolarization (MP in Figure 2) that only depends on parasympathetic but not sympathetic activity.

Based on these assumptions, heart period (HP) and heart rate (HR) can be calculated according to equations (1) and (2) with tsys: time from beginning of Phase 0 to end of Phase 3 in ms; MP: membrane potential at the end of Phase 3 repolarization in mV; *m*: slope of Phase 4 depolarization in mV/ms; th: threshold membrane potential for a new action potential in mV.

(1) $$\mathrm{HP}\;\left(\mathrm{ms}\right)=\text{tsys}+\frac{\mathrm{th}-\mathrm{MP}}{m},$$

(2) $$\mathrm{HR}\;\left(\mathrm{bpm}\right)=\frac{60,000}{\text{tsys}+\frac{\mathrm{th}-\mathrm{MP}}{m}}.$$

Considering fluctuations in MP (ΔMP, in mV) and fluctuations in m (Δm, in mV/ms), the amplitude of heart period (ΔHP) and heart rate (ΔHR) variability (difference from highest to lowest heart period or heart rate values) can be calculated according to equations (3) and (4):

(3) $$∆\mathrm{HP}=\frac{\mathrm{th}-\mathrm{MP}-∆\mathrm{MP}}{m+∆m}-\frac{\mathrm{th}-\mathrm{MP}+∆\mathrm{MP}}{m-∆m},$$

(4) $$∆\mathrm{HR}=\frac{60,000}{\text{tsys}+\frac{\mathrm{th}-\mathrm{MP}-∆\mathrm{MP}}{m+∆m}}-\frac{60,000}{\text{tsys}+\frac{\mathrm{th}-\mathrm{MP}+∆\mathrm{MP}}{m-∆m}}.$$

#### 2.1.1 Autonomic modulation of low frequency and high frequency variability of HP and HR

**FIGURE 3:**
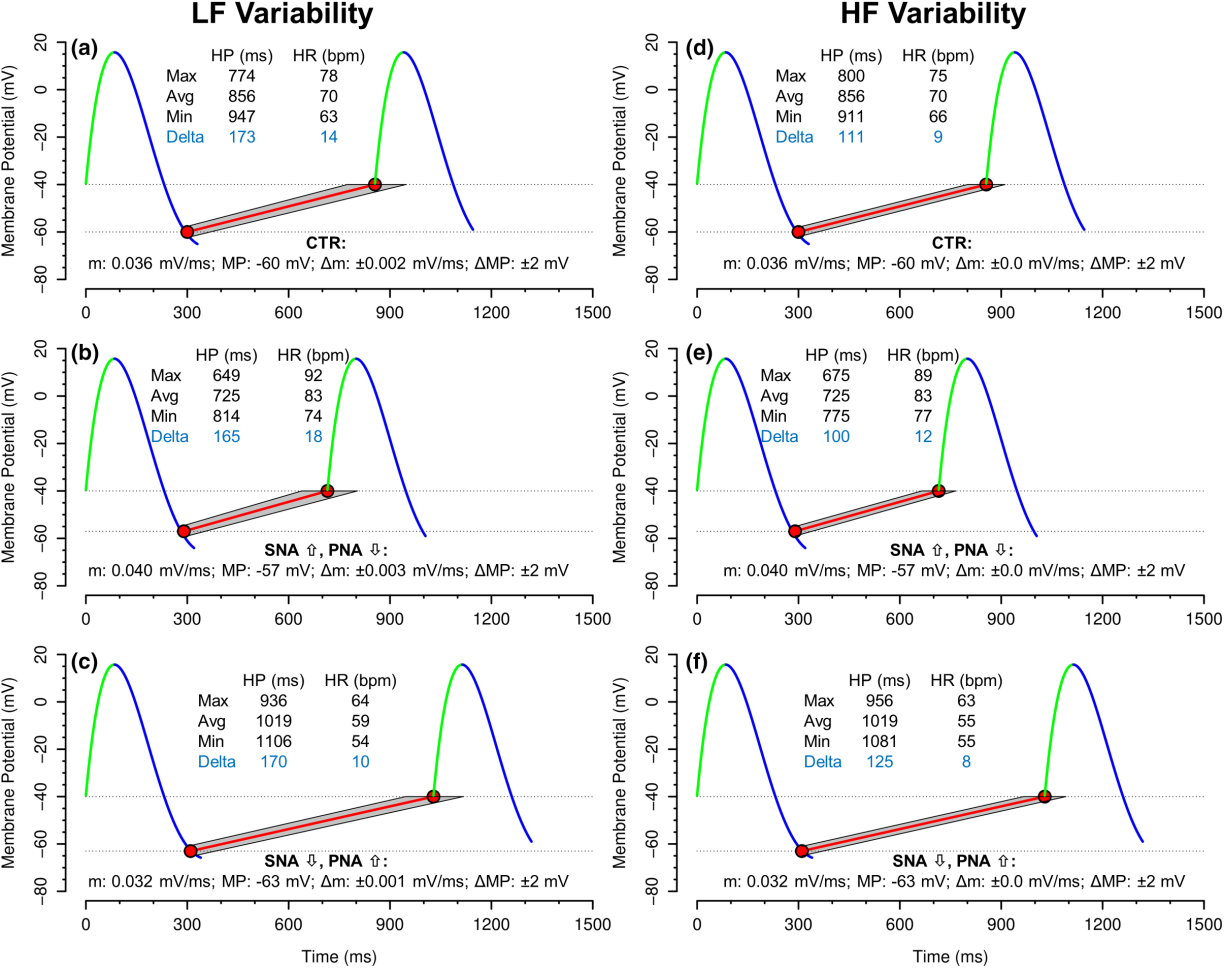
Effects of autonomic modulation of sinoatrial node function on low frequency (LF, panels a, b, and c) and high frequency (HF, panels d, e, and f), heart period (HP), and heart rate (HR) variability. Color coding represents the ion currents shown in Figure 1, with calcium current (*I*_Ca_) in green, potassium current (*I*_K_) in blue, and funny current (*I*_f_) in red. Panels a and d represent control conditions (CTR) with resting autonomic tone. Panels b and e represent increased sympathetic (SNA) and decreased parasympathetic nervous system activity (PNA). Panels c and f represent decreased SNA and increased PNA. The slope (*m*) of Phase 4 depolarization was defined as 0.036 mV/ms under resting conditions and increased to 0.040 mV/ms for increased SNA/decreased PNA and decreased to 0.032 mV/ms for decreased SNA/increased PNA. Membrane potential at the end of Phase 3 repolarization (MP) was set at −60 mV for control conditions and decreased to −57 mV and increased to −63 mV for decreased and increased PNA, respectively. Fluctuations in the slope of Phase 4 depolarization (Δm) were set to ±0.002 mV/ms for control conditions and increased to ±0.003 mV/ms for increased SNA/decreased PNA and reduced to ±0.001 mV/ms for decreased SNA/increased PNA. Since fluctuations in intracellular cAMP are too sluggish to respond at HF, Δm was set to 0.0 mV/ms for HF variability. Fluctuations in the MP at the end of Phase 3 repolarization (ΔMP) were set at ±2 mV for all conditions. Insets show the average (Avg) HP and HR, minimum (Min) and maximum (Max) deviation in HP and HR, and the variability (Delta values) in HP and HR associated with each condition.

In Figure 3, we have applied equations ((1), (2), (3), (4)) to illustrate the effects of changes in autonomic tone on LF and HF variability of HP and HR. We assumed that sympathetic (SNA) and parasympathetic nervous system activity (PNA) change reciprocally, that is, an increase in sympathetic tone is associated with a decrease in parasympathetic tone, and vice versa. The specific data used for the model shown in Figure 3 are provided within the figure and its legend. To simulate the effect of an increase in SNA and decrease in PNA on LF variability (Figure 3b), we increased the slope m from 0.036 to 0.040 mV/ms and decreased MP from −60 mV to −57 mV. We also raised the variability of m (Δm) from ±0.002 to ±0.003 mV/ms. It is important to note that this increase in Δm results from changes in the mean level of autonomic nervous system activity, even without changes in the variability of autonomic nervous system activity, because an increase in the mean level of SNA and a decrease in the mean level of PNA would increase the activity of adenylyl cyclase which would amplify existing fluctuations in cAMP (see Figure 1b). However, we left the fluctuations in MP (ΔMP) constant at ±2 mV, because ΔMP would only change if PNA variability would change but would not be affected by changes in the mean level of PNA. Reciprocal changes were made to simulate the effect of a decrease in SNA and an increase in PNA on LF variability (Figure 3c). To simulate the effect of changes in autonomic nervous system activity on HF variability (Figure 3d–f) we altered the slope m and membrane potential MP as with LF variability. However, we eliminated fluctuations in slope m (Δm = ±0.0 mV/ms) because slope m is too sluggish to follow HF variability. Thus, all HF variabilities of HP or HR solely depend on fluctuations in MP (ΔMP) that were left constant at ±2 mV.

#### 2.1.2 Effects of variability in potassium current (I_K_ ) and funny current (I_f_) on variability of HP and HR

**FIGURE 4:**
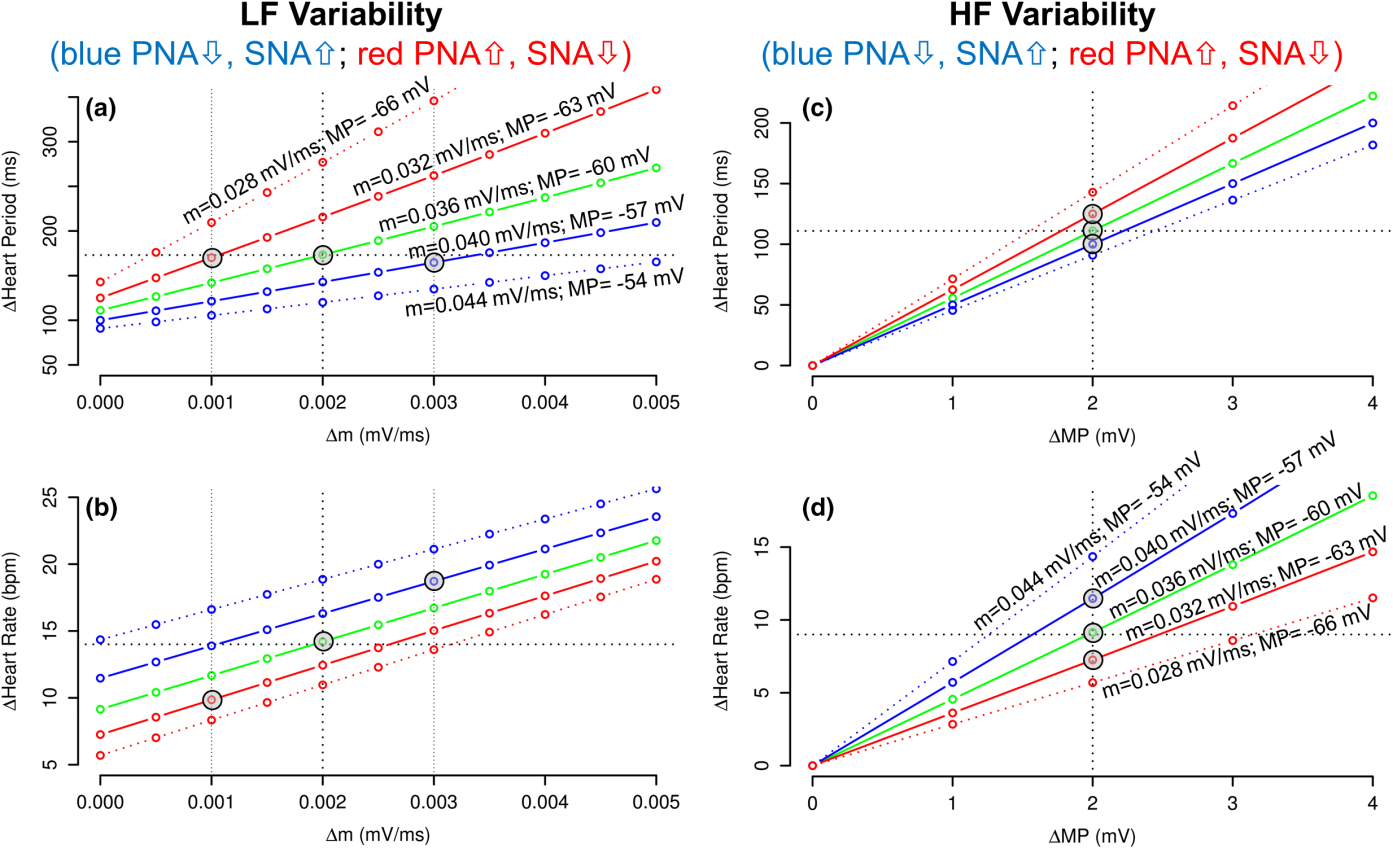
Effects of fluctuations in the slope of Phase 4 depolarization (Δ*m*) on low frequency (LF) heart period (panel a) and heart rate variability (panel b) and effects of fluctuations in membrane potential at the end of Phase 3 repolarization (ΔMP) on high frequency (HF) heart period (panel c) and heart rate variability (panel d). Green lines represent control conditions using the same parameters as that shown for the control condition in Figure 3a,b. Blue lines represent an increase in sympathetic nervous system activity (SNA) combined with a decrease in parasympathetic nervous system activity (PNA). Red lines represent an increase in PNA combined with a decrease in SNA. The solid lines are based on the same parameters as used for the respective condition in Figure 3. The gray circles represent the respective LF and HF variabilities (Delta values) from Figure 3. For LF variability, the fluctuations in the membrane potential at the end of Phase 3 repolarization (ΔMP) were set at ±2 mV. For HF variability, the fluctuations in the slope of Phase 4 depolarization (Δ*m*) was set at 0.0 mV/ms. The threshold membrane potential for triggering a new action potential was kept constant at −40 mV.

The potassium current I_K_ depends on PNA and determines the membrane potential at the end of Phase 3 repolarization (MP). Variability in PNA induces variability in *I*_K_ and, thus, variability in MP (ΔMP). The slope of Phase 4 depolarization (*m*) depends on the funny current *I*_f_, which depends on SNA and PNA. Variability in SNA and PNA, therefore, induces variability in the slope of Phase 4 depolarization (Δm). In Figure 4, we used equations (3) and (4) to examine the effect of Δm (Figure 4a,b) and ΔMP (Figure 4c,d) on LF and HF variability of HP (ΔHP, Figure 4a,c) and HR (ΔHR, Figure 4b,d). The model parameters (tsys, th, *m*, and MP) from Figure 3 were used for the control condition (green lines in Figure 4), the increase in SNA and decrease in PNA (solid blue lines in Figure 4), and the decrease in SNA and increase in PNA (solid red lines in Figure 4). In addition, we included more extreme perturbations in autonomic tone (dotted lines in Figure 4). To assess the effects of variability in I_f_ on LF variability (Figure 4a,b), we varied Δ*m* from ±0.000 to ±0.005 mV/ms. To assess the effects of variability in *I*_K_ on HF variability (Figure 4c,d), we varied ΔMP from ±0 to ±4 mV.

### 2.2 Effects of taVNS and OA‐D on HRV

Data from two separate studies were combined for this study. Both studies utilized the same experimental protocol, with the exception that in one study saliva samples were obtained (Kania et al., 2021) whereas in the other study (ongoing and unpublished) blood samples were obtained. Both studies were approved by the Institutional Review Board at Burrell College of Osteopathic Medicine (IRB# 0046_2019 and IRB# 0054_2019). The ongoing study is registered with ClinicalTrials.gov (NCT04177264). All study participants provided written informed consent prior to participating in the studies.

#### 2.2.1 Subjects

A total number of 49 (33 female and 16 male) adult generally healthy subjects participated in the study. Nineteen subjects were from the previously published study (Kania et al., 2021), and 30 subjects were from the ongoing study. The average age was 54.3 ± 16.4 years (mean ± SD), and the average body mass index (BMI) was 28.2 ± 6.5 kg/m^2^ (mean ± SD). Subjects were asked to continue their regular daily routine while in the research study. We did not ask subjects to abstain from exercise or caffeinated beverages. Throughout the study, subjects continued to take their medications as prescribed. Our laboratory is located outside of Las Cruces, NM, requiring subjects to drive to the laboratory, which takes a minimum of 30 min. At the beginning of each study day, we recorded body core temperature, upper arm blood pressure, body weight, and height, which took at least another 30 min. Thus, we are certain that subjects had not engaged in exercise or consumed caffeine for a minimum of 1 h prior to the experiments.

#### 2.2.2 Experimental protocol

**FIGURE 5:**
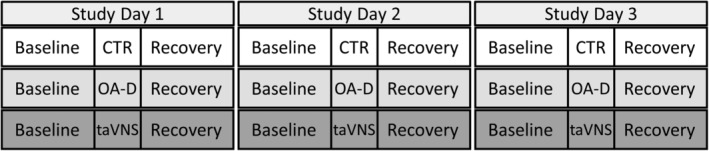
*Experimental Protocol*. Subjects (*n* = 49) were randomized in either the control group (CTR, *n* = 23, white background), the occipitoatlantal decompression group (OA‐D, *n* = 14, gray background), or the transcutaneous auricular vagus nerve stimulation group (taVNS, *n* = 12, dark gray background). Subjects reported to the laboratory on 3 consecutive study days. Depending on group assignment, the same intervention (CTR, OA‐D, or taVNS) was repeated on each of the 3 consecutive study days. Thus, each study participant only received either the CTR intervention, the OA‐D intervention, or the taVNS intervention. On each study day, a baseline recording of 30 min was followed by a 15 min intervention (CTR, OA‐D, or taVNS) and a final 30 min recovery recording.

The experimental protocol is described in detail in a previous publication (Kania et al., 2021) and illustrated in Figure 5. Subjects were randomized into the control group (*n* = 23, 18 female and 5 male), the occipitoatlantal decompression (OA‐D) group (*n* = 14, 8 female and 6 male), and the transcutaneous auricular vagus nerve stimulation (taVNS) group (*n* = 12, 7 female and 5 male). By study design, we are currently recruiting 4 times more subjects for the control group than for the OA‐D and taVNS groups, which explains the larger number of subjects in the control group. The subject characteristics including age, BMI, medical conditions, and medications for the three groups are summarized in Table 1. The medical conditions and medications are strictly based on what subjects reported in a study entry questionnaire and may be subject to recall bias. There were no significant differences in age or BMI between groups. The low numbers of subjects with different medical conditions and medications do not allow for a meaningful statistical analysis. But overall, the control group appears to be using a wider range of medications compared to the OA‐D and taVNS groups. Each subject participated in 3 consecutive study days (e.g., Monday, Tuesday, and Wednesday). For individual subjects, the experimental protocol and intervention (i.e., control, OA‐D, or taVNS) were the same on each of the 3 consecutive study days. Thus, an individual subject exclusively received either the control intervention, the OA‐D intervention, or the taVNS intervention, depending on group assignment (Figure 5). Some subjects reported to the laboratory in the morning at 8:00 am, and others reported to the laboratory in the afternoon typically at 2:00 pm. However, individual subjects reported to the 3 consecutive study days always at the same time of the day. During the experimental protocol, subjects were lying in the supine position on an OMT examination table. Subjects were instrumented with EKG electrodes and a blood pressure sensor (Finapres Finometer Pro; Finapres Medical Systems) on a finger of the left hand. The blood pressure signal was not used for the current study. An initial baseline recording of 30 min duration was followed by an intervention that consisted of 15 min rest (control group), 5 min (in previously published study), or 10 min (in ongoing unpublished study) of OA‐D followed by 10 or 5 min of rest for a 15 min intervention (OA‐D group), or 15 min of taVNS (taVNS group). The intervention was followed by 30 min of recovery recording (Figure 5).

**TABLE 1:** Subject characteristics.

Parameter	Control	OA‐D	taVNS
Number of subjects	23	14	12
Sex (female/male)	18/5	8/6	7/5
Age (years, mean ± SD)	56.0 ± 17.2	50.6 ± 17.5	55.3 ± 13.7
BMI (kg/m^2^, mean ± SD)	26.8 ± 5.5	28.6 ± 3.9	30.4 ± 9.7
Medical conditions (number of subjects)			
Hypertension	4	1	1
Coronary artery disease	1	0	0
Depression/Anxiety	1	1	3
Allergies	0	0	1
Dermatologic conditions	1	0	1
Thyroid conditions	2	0	0
GI condition	0	2	0
Medications (number of subjects)			
Vitamins	11	2	6
RAS inhibitors	5	0	1
CCBs	1	0	0
Diuretics	3	0	0
Statins/PCSK9‐inhibitors	4	0	0
Aspirin	1	0	0
Antidepressants/Anxiolytics	2	2	5
Topical steroids	4	1	0
GI drugs	3	1	1
Antihistamines (H1‐blocker)	1	0	1
Spasmolytic drug (tizanidine)	1	0	0
Levothyroxine	1	0	0

*Note*: Renin–angiotensin system (RAS) inhibitors include angiotensin AT1‐receptor blockers and converting enzyme inhibitors. CCBs: calcium channel blockers. Antidepressants/anxiolytics include selective serotonin reuptake inhibitors, bupropion, and gabapentin. PCSK9: proprotein convertase subtilisin kexin type 9. GI drugs include digestive enzymes, proton pump inhibitors, and histamine H2‐blockers.

#### 2.2.3 Occipitoatlantal decompression (OA‐D)

It is an osteopathic technique that focuses on treating an articular compression between the occiput and the atlas, which is thought to improve conditions relating to the path of the vagus nerve as it exits the skull. This technique has been demonstrated to increase HF HRV (Giles et al., 2013). OA‐D was performed by either an osteopathic physician or well‐trained osteopathic medical students. For OA‐D, the investigator cradled the subjects’ heads with their hands and finger pads along the inferior aspect of inion, reaching toward the occipitoatlantal joint. The investigator then applied gentle anterior and cephalad traction to the occiput, while bringing the elbows together. This motion resulted in supination of the hands and separation of the fingers, creating an anterolateral force vector to either side of the foramen magnum (DeStefano, 2011). The gentle anterior and cephalad traction was then maintained for 5 min in the previously published study (Dalgleish et al., 2021; Kania et al., 2021) or for 10 min in the ongoing unpublished study.

#### 2.2.4 Transcutaneous auricular vagus nerve stimulation (taVNS)

A bipolar clip electrode connected to a transcutaneous electrical nerve stimulator (EMS 7500; Current Solutions, LLC) was applied to the cymba conchae that is innervated by the auricular branch of the vagus nerve (Arnold's nerve) (He et al., 2012). The clip electrode was applied to the ear lobe such that the cathode was placed at the cavum of the concha and the anode was placed at the opposing site of the back of the auricle that is also innervated by the vagus nerve. The stimulation parameters were 10 Hz stimulation frequency and 300 μs pulse width. The stimulation current was determined individually for each subject by slowly increasing the stimulation current until the subjects felt a mild tingling sensation at the site of the electrode. Then, the current was gradually reduced until the tingling sensation disappeared or was just barely felt. This current was then used for 15 min of taVNS.

#### 2.2.5 Data analysis

Data analysis was conducted using the freely available HemoLab software (Stauss, 2008). Beat‐by‐beat heart rate time series were extracted from a total number of 441 EKG recordings (49 subjects, 3 study days, baseline, intervention, and recovery). All heart rate time series were visually inspected, and artifacts or heart beats resulting from premature ventricular contractions were replaced with interpolated values based on leading and trailing values, which affected less than 1% of data points. Beat‐by‐beat heart rate time series were converted to RR‐interval (heart period) time series. RR‐interval time series were used to calculate the standard deviation of all normal RR‐intervals (SDNN) and the root mean square of successive differences between normal heartbeats (RMSSD). For this, the beat‐by‐beat RR‐interval time series were divided into 50% overlapping segments of 5 min duration each. SDNN and RMSSD were then calculated for each of the 5 min segments, and the SDNN and RMSSD values of all 5 min segments were averaged. These mean SDNN and RMSSD values were used for statistical analysis. For frequency‐domain HRV analysis, beat‐by‐beat heart rate and RR‐interval values were spline interpolated and resampled at an equidistant sampling rate of 12 Hz. From these 12 Hz time series, power spectra were calculated for overlapping segments (50% overlap, 2024 data points, 169 s). The power spectra of the overlapping segments for each subject and each experimental condition (baseline, intervention, recovery) were averaged. Low frequency (LF) and HF spectral powers were calculated as the area under the curve of the power spectra in the frequency bands of 0.04–0.15 Hz and 0.15–0.4 Hz, respectively as suggested by the Task Force of the European Society of Cardiology and the North American Society of Pacing and Electrophysiology (Task Force of the European Society of Cardiology and the North American Society of Pacing and Electrophysiology, 1996). Absolute spectral powers with units of ms^2^ for heart period variability and bpm^2^ for heart rate variability were used for statistical analysis.

#### 2.2.6 Statistical analysis

Statistical analysis was performed using the WinStat software included with the HemoLab software (Stauss, 2008). Throughout the text of the manuscript and in the table, data are shown as means±standard deviation (SD). In figures, data are provided as means±standard error of the mean (SEM). For statistical comparison between groups, one‐way analysis of variance (ANOVA) for independent measures was used. Post hoc Fisher tests were used if the ANOVA revealed statistical significance to identify differences between individual groups. For statistical comparison between data obtained on the three study days or between data obtained during the three phases of the experimental protocol (baseline, intervention, and recovery), repeated‐measures one‐way ANOVA was used. In case of statistical significance in the ANOVA, post hoc Fisher tests were used to identify significant differences between individual study days or protocol phases.

## 3 RESULTS

### 3.1 Model simulations

Based on the electrophysiologic phenomena driving sinus node automaticity, we developed a model for the prediction of LF and HF heart rate (HR) and heart period (HP) variability in response to changes in sympathetic (SNA) or parasympathetic nervous system activity (PNA). The model assumes that LF variability of HR or HP depends on variability in the membrane potential at the end of Phase 3 repolarization and in variability of the slope of Phase 4 depolarization (Figures 1 and 2). In contrast, HF variability of HR or HP only depends on variability in the membrane potential at the end of Phase 3 repolarization, because fluctuations in intracellular cAMP that determine fluctuations in the slope of Phase 4 depolarization are too sluggish to follow HF variability (Figures 1 and 2). We then applied the model to changes in SNA and PNA. For this, we assumed that SNA and PNA change reciprocally, that is, an increase in SNA is accompanied by a decrease in PNA and vice versa. Figure 3 shows the simulated effects of alterations in SNA and PNA on LF and HF variability of HP and HR. For this simulation, we altered the mean level (tone) of SNA and PNA, but we left the amplitude of spontaneous variability of SNA and PNA around the mean level constant. Thus, fluctuations of the membrane potential at the end of Phase 3 repolarization (ΔMP in Figure 3) were kept constant at ±2 mV. However, changes in the mean levels of SNA and PNA will alter the activity of adenylyl cyclase (Figure 1b). Thus, even if the variability of SNA and PNA is constant, LF variability of the intracellular cAMP concentration will change if the mean level of SNA or PNA changes. Therefore, for the simulation of LF variability we increased the variability of the slope of Phase 4 depolarization (Δ*m*, in Figure 3a) from ±0.002 to ±0.003 mV/ms with increased SNA and decreased PNA (Figure 3b, increased activity of adenylyl cyclase) and decreased Δm from ±0.002 to ±0.001 mV/ms with decreased SNA and increased PNA (Figure 3c, decreased activity of adenylyl cyclase). For the simulation of HF variability, we removed the variability of Δ*m* by setting it to ±0.0 mV/ms (Figure 3d–f), because the generally accepted assumption is that fluctuations in cAMP are too sluggish to follow HF variability (Stauss et al., 1997).

Based on these considerations, our model simulation showed that LF variability of HP decreased from 173 ms (±86.5 ms) under control conditions (Figure 3a) to 165 ms (±82.5 ms) with increased SNA and decreased PNA (Figure 3b) and to 170 ms (±85 ms) with decreased SNA and increased PNA (Figure 3c). In contrast, LF variability of HR increased from 14 bpm (±7 bpm) under control conditions (Figure 3a) to 18 bpm (±9 bpm) with increased SNA and decreased PNA (Figure 3b) and decreased to 10 bpm (±5 bpm) with decreased SNA and increased PNA (Figure 3c). Thus, only LF variability of HR (in bpm), but not LF variability of HP (in ms), is consistent with the generally accepted notion that elevated sympathetic tone is associated with high LF variability.

The model simulation also showed that HF variability of HP decreased from 111 ms (±55.5 ms) under control conditions (Figure 3d) to 100 ms (±50.0 ms) with decreased PNA and increased SNA (Figure 3e) and increased to 125 ms (±62.5 ms) with increased PNA and decreased SNA (Figure 3f). In contrast, HF variability of HR increased from 9 bpm (±4.5 bpm) under control conditions (Figure 3d) to 12 bpm (±6.0 bpm) with decreased PNA and increased SNA (Figure 3e) and decreased to 8 bpm (±4 bpm) with increased PNA and decreased SNA (Figure 3f). Thus, only HF variability of HP (in ms), but not HF variability of HR (in bpm), is consistent with the generally accepted notion that elevated parasympathetic tone is associated with high HF variability.

To further investigate these relationships, we simulated LF variability of HP and HR (Figure 4a,b) in response to changes in the variability of the slope of Phase 4 depolarization (Δ*m*) and HF variability of HP and HR (Figure 4c,d) in response to changes in the variability of the membrane potential at the end of Phase 3 repolarization (ΔMP). For these simulations we modulated autonomic tone. For an increase in SNA with associated decrease in PNA (blue lines in Figure 4), we increased the slope of Phase 4 depolarization (*m*) and decreased (less negative) the membrane potential at the end of Phase 3 repolarization (MP). For an increase in PNA with associated decrease in SNA (red lines in Figure 4), we reduced the slope of Phase 4 depolarization (*m*) and increased (more negative) the membrane potential at the end of Phase 3 repolarization (MP). These simulations revealed that an increase in sympathetic tone (with associated decrease in parasympathetic tone, blue lines in Figure 4) decreased LF variability of HP (in ms) but increased LF variability of HR (in bpm) compared to control conditions (green line). Likewise, a decrease in sympathetic tone (with associated increase in parasympathetic tone, red lines in Figure 4) increased LF variability of HP (in ms) but decreased LF variability of HR (in bpm) compared to control conditions. Thus, only LF variability of HR (in bpm) is consistent with the notion that increased sympathetic tone is associated with increased LF variability. An increase in LF variability of HP (in ms) would indicate an increase in parasympathetic tone and/or a decrease in sympathetic tone.

The simulation shown in Figure 4 also reveals that elevated parasympathetic tone (with associated decrease in sympathetic tone, red lines in Figure 4) is associated with increased HF variability of HP (in ms) but decreased HF variability of HR (in bpm). Likewise, a decrease in parasympathetic tone (with associated increase in sympathetic tone, blue lines in Figure 4) is associated with a decrease in HF variability of HP (in ms), but with an increase in HF variability of HR (in bpm). Thus, only HF variability of HP (in ms), but not HF variability of HR (in bpm), is consistent with the notion that elevated parasympathetic tone is associated with an increase in HF variability.

Taken together, our model simulation seems to suggest that autonomic modulation of LF variability of sinus node automaticity is best studied using HR time series in bpm, while autonomic modulation of HF variability of sinus node automaticity is best studied using HP time series in ms. Based on these considerations, the LF to HF ratio that is often considered a measure of autonomic balance (Task Force of the European Society of Cardiology and the North American Society of Pacing and Electrophysiology, 1996) may be most reliable when calculated as the ratio of LF variability of HR (in bpm) to HF variability of HP (in ms).

### 3.2 Effects of taVNS and OA‐D on HR and HRV

**FIGURE 6:**
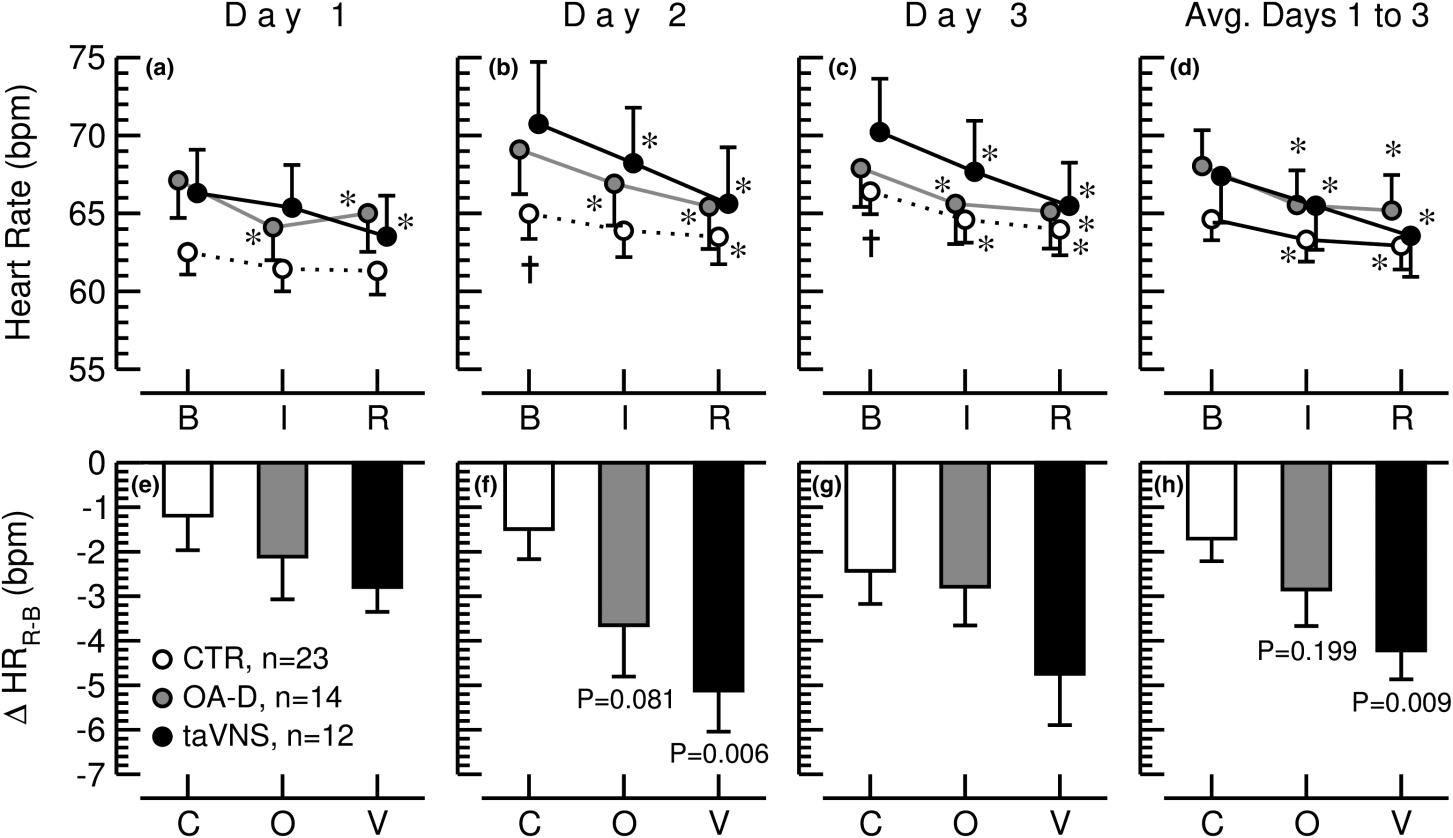
*Panels a–d (top)*: Heart rate during the 30‐min baseline recording (b), the 15‐min control (CTR, white circles, *n* = 23), occipitoatlantal decompression (OA‐D, gray circles, *n* = 14), or transcutaneous auricular vagus nerve stimulation (taVNS, black circles, *n* = 12) interventions (I), and during the 30‐min recovery recording (R) on 3 consecutive days and for the values from all 3 days averaged (Avg. Days 1 to 3). Heart rate was derived from the EKG. Data are means ± SEM. †*p* < 0.05 (repeated‐measures ANOVA) for baseline values on Days 2 or 3 versus baseline values on Day 1. *p < 0.05 (repeated‐measures ANOVA) for values during the intervention (I) or during the recovery period (R) compared to baseline values (B). *Panels e–h (bottom)*: Change in heart rate from baseline to recovery (Δ HR_R‐B_) for the control (C, *n* = 23), occipitoatlantal decompression (O, *n* = 14), and taVNS (V, *n* = 12) interventions on 3 consecutive days and for the values from all 3 days averaged (Avg. Days 1–3). Heart rate was derived from the EKG. Data are means ± SEM. P‐values (independent measures ANOVA) are for occipitoatlantal decompression (O, *n* = 14) or taVNS (V, *n* = 12) compared to the control (C, *n* = 23) intervention. For Days 1 and 3, the overall ANOVA was not significant and, therefore, no post hoc tests were performed, and no *p*‐values are provided.

Figure 6 shows the HR response to the control, the OA‐D, and the taVNS interventions on the 3 consecutive study days as well as for the data from the three study days averaged. Throughout the experimental protocol HR declined for all three interventions and except for the control conditions on day one, this reduction in heart rate was significant (*p* < 0.05) for all conditions, all study days, and for the data from all 3 days averaged (Figure 6a–d). However, the decrease in heart rate from baseline to recovery was significantly greater for the taVNS intervention (−4.2 ± 2.2 bpm) than for the control intervention (−1.7 ± 2.4 bpm, *p* < 0.05) when the data from all three study days were averaged (Figure 6h). The heart rate responses to the control and OA‐D interventions were not significantly different. These findings suggest that taVNS exerts a greater autonomic effect (increased PNA and/or decreased SNA) compared to the control or OA‐D interventions.

**FIGURE 7:**
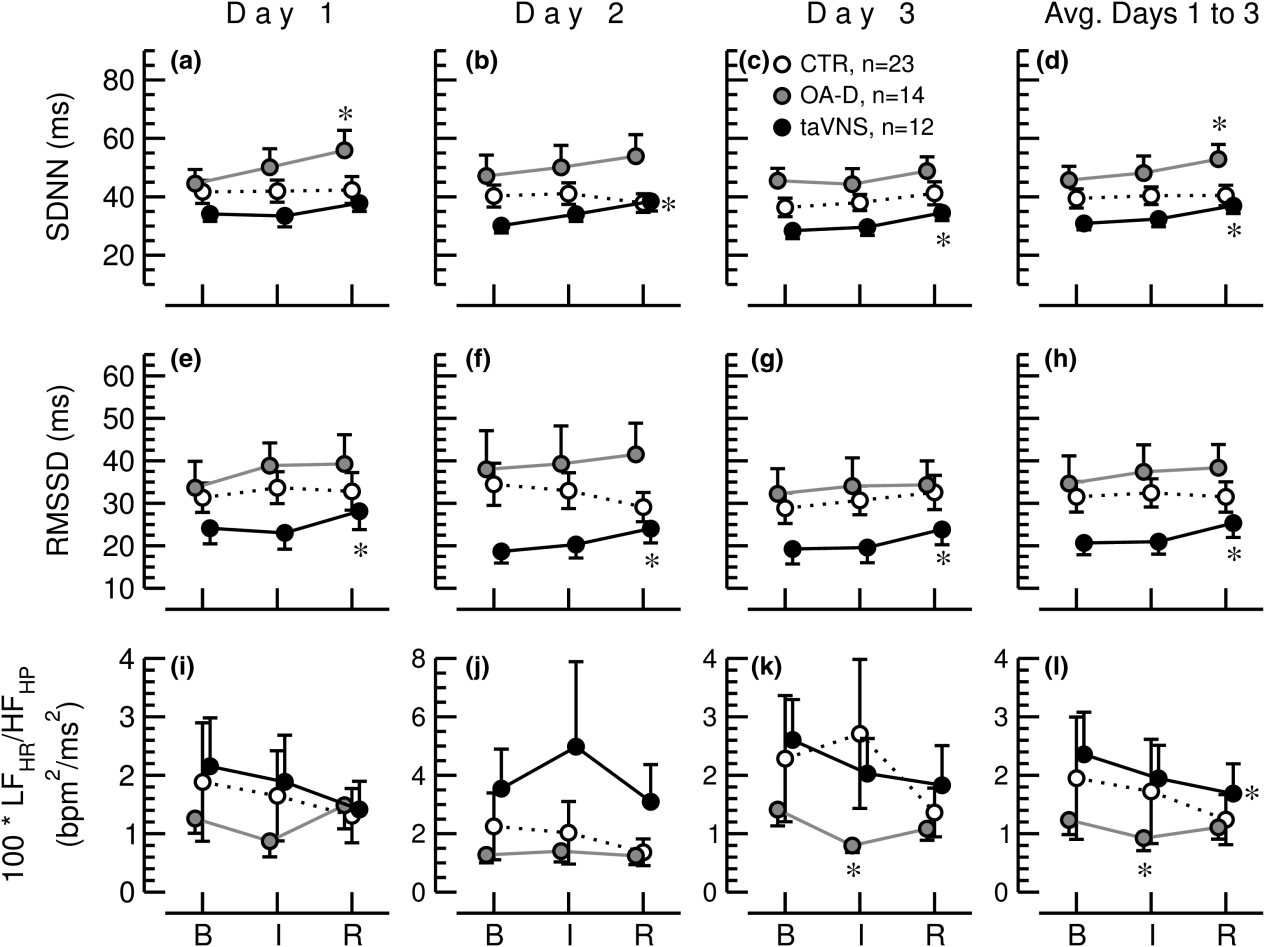
Standard deviation of all NN intervals (SDNN, panels a–d) and square root of the mean of the sum of the squares of differences between adjacent NN intervals (RMSSD, panels e–h), and low frequency (LF, calculated from heart rate time series) to high frequency (HF, calculated from heart period time series) ratio (LF_HR_/HF_HP_, panels i–l) during the 30‐min baseline recording (B), the 15‐min control (CTR, white circles, *n* = 23), occipitoatlantal decompression (OA‐D, gray circles, *n* = 14), or transcutaneous auricular vagus nerve stimulation (taVNS, black circles, *n* = 12) interventions (I), and during the 30‐min recovery recording (R) on 3 consecutive days and for the values from all 3 days averaged (Avg. Days 1–3). Data are means ± SEM. *p < 0.05 (repeated‐measures ANOVA) for values during the intervention (I) or during the recovery period (R) compared to baseline values (B).

Overall HR variability assessed by the time‐domain parameter SDNN generally increased with OA‐D and taVNS, but not with the control intervention (Figure 7a–d). This increase in SDNN with OA‐D and taVNS was statistically significant when the data from all three study days were averaged (Figure 7d). RMSSD is another time‐domain variability parameter that assesses beat‐by‐beat fluctuations of successive heart periods. As such, it is reflective of HF variability of HP. The taVNS intervention significantly increased RMSSD from baseline to the recovery recording on all three study days and when all three study days were average (Figure 7e–h). No significant effects of the control intervention or OA‐D on RMSSD were observed.

**FIGURE 8:**
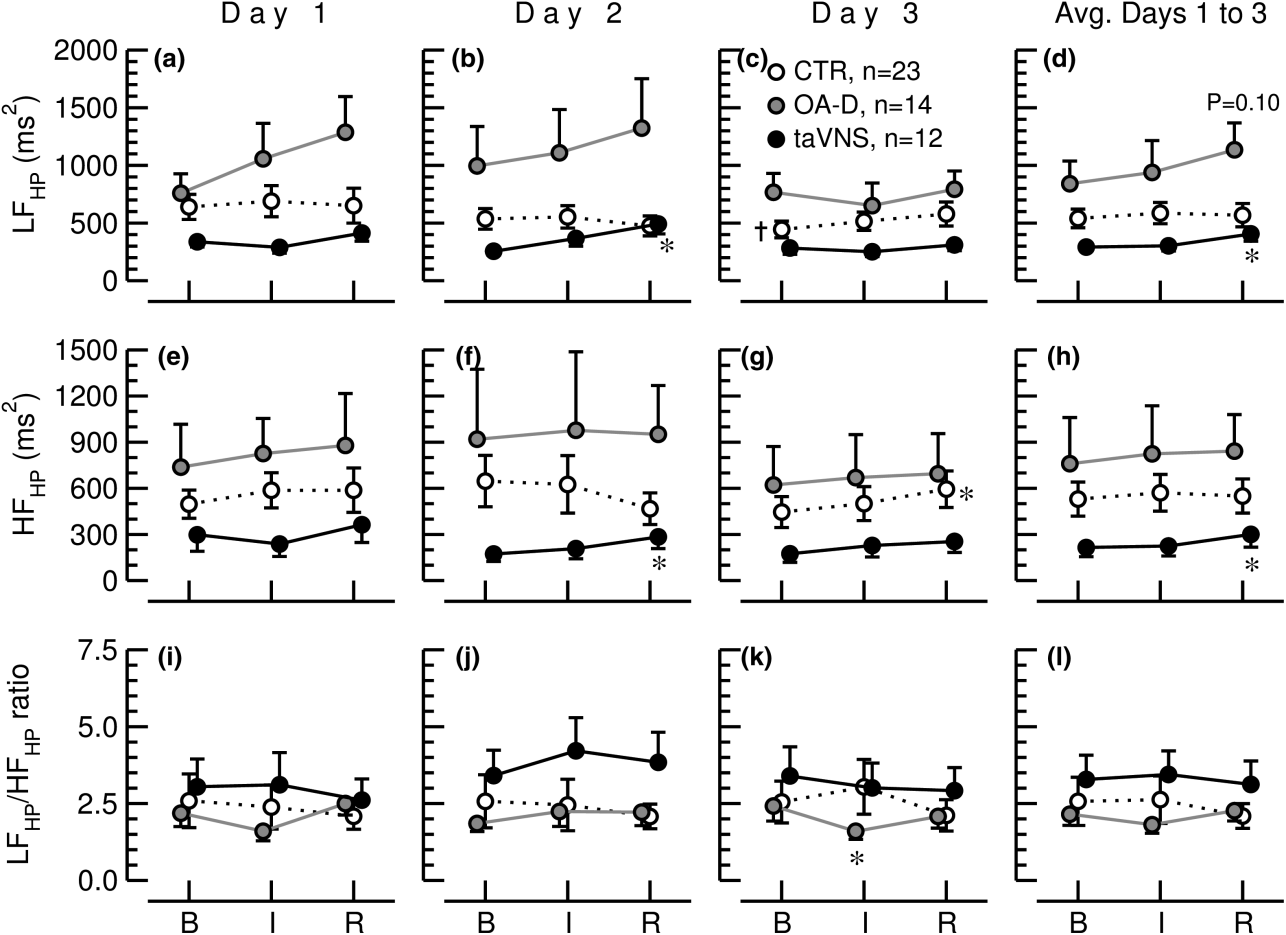
Low frequency (LF_HP_, panels a–d) and high frequency (HF_HP_, panels e–h) spectral powers of heart periods and LF to HF ratio of heart periods (LF_HP_/HF_HP_ ratio, panels i–l during the baseline recording (B), the control (CTR, white circles, *n* = 23), occipitoatlantal decompression (OA‐D, gray circles, *n* = 14), or transcutaneous auricular vagus nerve stimulation (taVNS, black circles, *n* = 12) interventions (I), and during the 30‐min recovery recording (R) on 3 consecutive days and for the values from all 3 days averaged (Avg. Days 1–3). Data are means ± SEM. †*p* < 0.05 (repeated‐measures ANOVA) for baseline values on Days 2 or 3 versus baseline values on Day 1. *p < 0.05 (repeated‐measures ANOVA) for values during the intervention (I) or during the recovery period (R) compared to baseline values (B).

**FIGURE 9:**
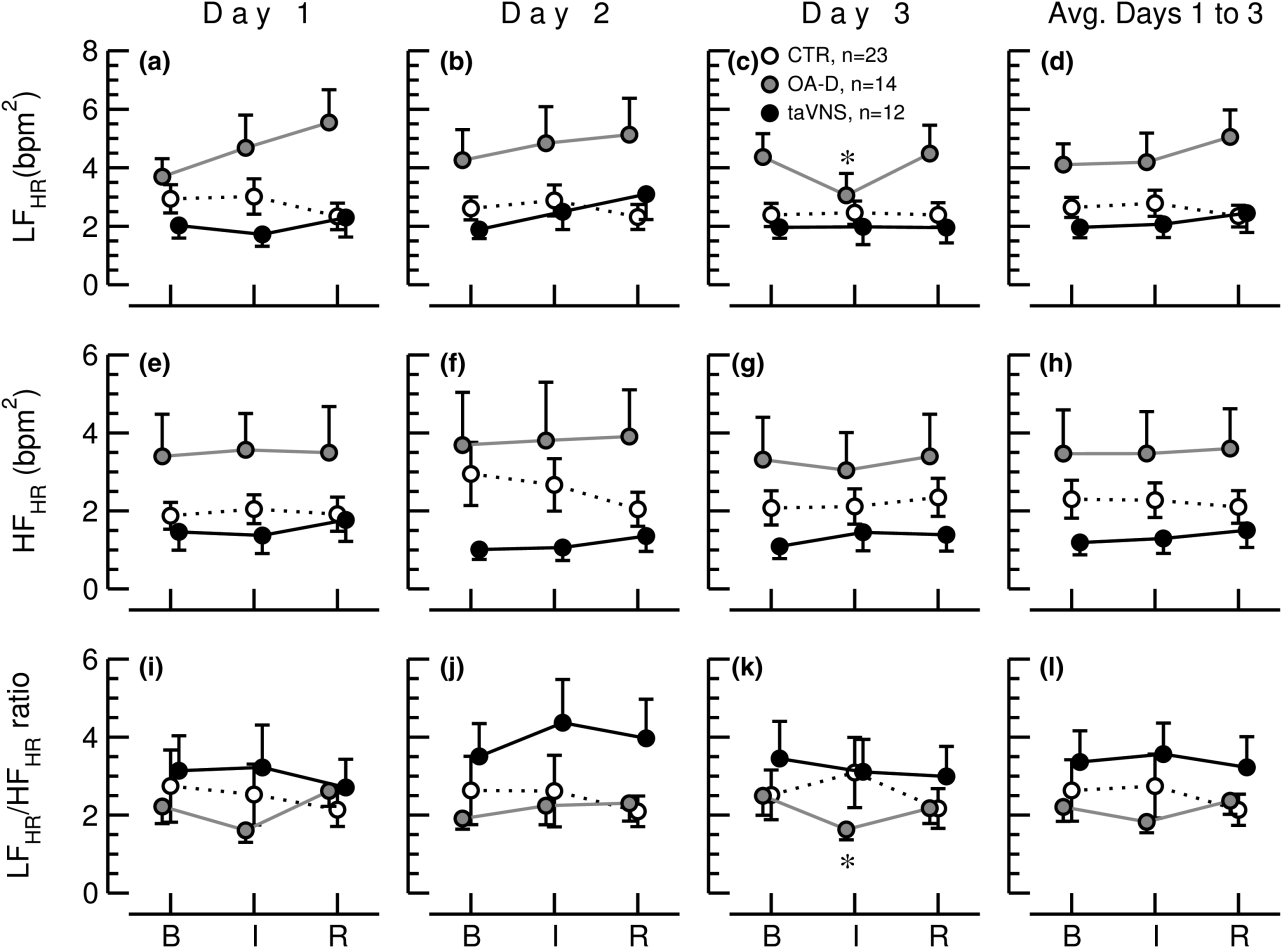
Low frequency (LF_HR_, panels a–d) and high frequency (HF_HR_, panels e–h) spectral powers of heart rate (HR) and LF_HR_ to HF_HR_ ratio of HR (LF_HR_/HF_HR_ ratio, panels i–l) during the baseline recording (B), the control (CTR, white circles, *n* = 23), occipitoatlantal decompression (OA‐D, gray circles, *n* = 14), or transcutaneous auricular vagus nerve stimulation (taVNS, black circles, *n* = 12) interventions (I), and during the 30‐min recovery recording (R) on 3 consecutive days and for the values from all 3 days averaged (Avg. Days 1–3). Data are means ± SEM. *p < 0.05 (repeated‐measures ANOVA) for values during the intervention (I) or during the recovery period (R) compared to baseline values (B).

The results of power spectral analyses of HP and HR time series are presented in Figures 8 and 9, respectively. When the data from all three study days were averaged, LF variability of HP increased significantly from baseline to the recovery recording with the taVNS intervention, but not with the control or OA‐D interventions (Figure 8d). Likewise, with the data from all three study days averaged, HF variability of HP only increased significantly in response to the taVNS intervention (Figure 8h). For the control intervention, an increase in HF variability of HP was observed only on the third study day (Figure 8g), but that increase in HF variability of HP did not hold true when the data from all three study days were averaged. Except for a decrease during the OA‐D intervention on the third study day (Figure 8k), no significant effects of the control intervention, OA‐D, or taVNS on LF/HF ratio calculated from heart periods were observed (Figure 8i–l).

Interestingly, when the power spectral analysis is calculated from heart rate time series different results are obtained. It is important to point out that the heart period time series were directly derived from the heart rate time series by dividing 60,000 by the heart rate. Thus, identical time series expressed as either heart rate or heart period were used for power spectral analysis. When calculated from HR time series, LF variability was not significantly affected by any intervention, except a decrease in LF variability during the OA‐D intervention on the third study day (Figure 9a–d). Likewise, no significant effects of any of the interventions were seen for HF variability of HR (Figure 9e–h). For the LF/HF ratio calculated from HR time series, the only significant finding was a decrease during the OA‐D intervention on the third study day (Figure 9i–l).

## 4 DISCUSSION

The most important finding of this study is that it matters if HRV analysis is performed from heart rate (in bpm) or heart period (in ms) time series. For frequency‐domain analysis, LF variability reflects autonomic modulation more reliably when calculated from heart rate time series (in bpm), while HF variability better reflects autonomic modulation when calculated from heart period time series (in ms). This conclusion is based on model simulations of the effects of sympathetic and parasympathetic nervous system activity on automaticity of sinus node function. This simulation demonstrated that LF variability of heart period is reduced, rather than increased, when sympathetic activity increases and parasympathetic activity decreases (blue lines in Figure 4a are below the green line for the control condition). However, if expressed in units of heart rate (bpm), LF variability is increased, as expected, when sympathetic activity increases and parasympathetic activity decreases (blue lines in Figure 4b are above the green line for the control condition). The simulation for HF variability revealed that HF variability of heart period, as expected, is increased with increased parasympathetic and decreased sympathetic activity (red lines in Figure 4c are above the green line for the control condition). However, when expressed in units of heart rate (bpm), HF variability is reduced when parasympathetic activity increases and sympathetic activity decreases (red lines in Figure 4d are below the green line for the control condition). These simulations are based on the assumption that the mean level of autonomic nervous system activity changes without a change in the amplitude of fluctuations in autonomic activity around the respective mean level. If the amplitude of the fluctuations in autonomic activity around the mean level would change along with the mean level of autonomic activity, it is possible that frequency‐domain parameters calculated from heart rate and heart period would show similar trends. However, this may only happen with pronounced perturbations of autonomic nervous system activity, such as during head‐up tilt, or other autonomic maneuvers. More subtle changes in autonomic tone as would be expected from taVNS or OA‐D may not be associated with changes in the amplitude of the fluctuations in autonomic tone around the mean level.

The Task Force of The European Society of Cardiology and The North American Society of Pacing and Electrophysiology recommended calculating frequency‐domain HRV parameters from heart period time series in (ms) without providing a mechanistic rationale for this recommendation (Task Force of the European Society of Cardiology and the North American Society of Pacing and Electrophysiology, 1996). Instead, the task force referred to references in which autonomic tone was perturbed by maneuvers, such as head‐up tilt, a maneuver that likely did not only change the mean level of autonomic tone, but also affected the variability of autonomic nervous system activity. Another committee report discussed the issue of heart period versus heart rate in more detail (Berntson et al., 1997). In line with our finding, this committee stated that “heart period is more typically employed and is generally preferred, especially when the interest is in indexing parasympathetic control.” However, the committee also stated that “The issue is somewhat more complex for sympathetic contributions to lower frequency rhythms because functions relating the frequency of sympathetic neural activity are nonlinear for both heart rate and heart period.” (Berntson et al., 1997). Even though the committee report from 1997 (Berntson et al., 1997) is more cautious regarding a recommendation for the use of heart period for assessing sympathetic contributions to lower frequency rhythms, most investigators nowadays follow the earlier Task Force recommendations from 1996 (Task Force of the European Society of Cardiology and the North American Society of Pacing and Electrophysiology, 1996) and use heart period time series to assess sympathetic contributions to LF rhythms. Partly this practice may be due to convenience. Consistent with our model simulation, it is generally accepted that parasympathetic modulation of HF variability is most reliable when calculated from heart period time series. Assessing sympathetic modulation of LF variability also from heart period time series instead of from heart rate time series does not require performing a second power spectral analysis from heart rate time series. However, our simulation suggests that more reliable results can be obtained by using heart rate time series when assessing sympathetic modulation of LF variability.

Data analysis of EKG recordings before during and after OA‐D, taVNS, and a time control intervention on 3 consecutive days demonstrated that heart rate decreased throughout the experimental protocol in response to all three interventions (Figure 6a–d). This finding indicates that sympathetic tone decreased and/or parasympathetic tone increased throughout the experimental protocol. This autonomic response was stronger in response to taVNS compared to the control intervention because the decrease in heart rate from baseline to recovery was greater in response to taVNS than in response to the control intervention (Figure 6e–h). This finding is confirmed by time‐domain analysis that shows that RMSSD increased from baseline to the recovery recording only in response to the taVNS intervention but not in response to OA‐D or the control intervention (Figure 7e–h). Since RMSSD is calculated as the variability between consecutive heart beats, it mostly reflects HF variability that is mainly depending on the parasympathetic nervous system. Interestingly, when the data from all three study days were averaged, SDNN increased from baseline to recovery for the OA‐D and taVNS intervention, but not for the control condition (Figure 7d). SDNN is an overall HRV parameter that responds to alterations in sympathetic and parasympathetic tone, just as LF frequency‐domain variability. Since taVNS increased RMSSD, the increase in SDNN with taVNS most likely is the result of parasympathetic activation. However, since OA‐D did not affect RMSSD which reflects HF variability, one would assume that the increase in SDNN is related to an increase in LF variability. SDNN is calculated from heart period time series. Based on our model simulation (Figure 4a), an increase in LF variability of heart period is most likely caused by an increase in parasympathetic activity accompanied by a decrease in sympathetic activity (red lines in Figure 4a). However, since OA‐D did not affect RMSSD, the conclusion would be that the increase in SDNN with OA‐D is caused by a decrease in sympathetic activity with no significant change in parasympathetic activity. This interpretation is in line with the significant decrease in heart rate in response to OA‐D (Figure 6a–d).

Frequency‐domain analysis showed that HF variability of HP calculated from RR‐interval time series averaged over all three study days increased only with taVNS but not in response to OA‐D or the control intervention (Figure 8h). Consistent with our model simulation (Figure 4c), this finding indicates that taVNS increases parasympathetic tone and possibly reduces sympathetic tone. Interestingly, LF variability of HP calculated from RR‐intervals also increased significantly in response to taVNS (Figure 8d). Based on our model simulation (Figure 4a, red lines), this finding would be best explained by an increase in parasympathetic nerve activity possibly accompanied by a decrease in sympathetic activity, which is consistent with the increase in HF variability of HP and the increase in RMSSD in the time‐domain analysis. For OA‐D, no significant changes in LF or HF variability were observed when calculated from RR‐intervals, although there was a trend toward an increase in LF variability of HP (*p* = 0.10 for the three study days averaged, Figure 8d). Consistent with our model simulation (Figure 4a, red lines), this trend would suggest a decrease in sympathetic nervous system activity in response to OA‐D with no significant change in parasympathetic activity, because neither RMSSD nor HF variability of HP was affected by OA‐D. Except a decrease with OA‐D on Day 3 (Figure 8k), no significant changes in LF/HF ratio calculated from RR‐intervals were observed (Figure 8i–l). Based on our model simulation, this finding is not surprising, because LF and HF variabilities of HP increase with increased parasympathetic and decreased sympathetic tone (red lines in Figure 4a,c), resulting in no change in LF/HF ratio.

When the frequency‐domain analysis was computed using heart rate time series, no significant effects of taVNS or the control intervention were observed. For OA‐D, a decrease on LF variability of HR and a decrease in LF/HF ratio were observed only on the third study day. According to our model simulation (red lines in Figure 4b), a decrease in LF variability of HR can best be explained by an increase in parasympathetic and decrease in sympathetic tone. However, since OA‐D did not affect RMSSD or HF variability of HP, the most likely conclusion is that OA‐D decreased sympathetic tone with no significant change in parasympathetic activity. This conclusion is consistent with the time‐domain analysis and the trend in the frequency‐domain analysis computed from RR‐intervals.

Based on our model simulation, LF and HF variabilities respond in the same direction to changes in autonomic tone when they are calculated from HP (Figure 4a vs. Figure 4c) or HR time series (Figure 4b vs. Figure 4d). Thus, the effects of changes in autonomic tone on the LF/HF ratio may cancel out. Therefore, we propose to calculate the LF/HF ratio from LF spectral power calculated from HR (LF_HR_) time series and HF spectral powers calculated from heart period time series (HF_HP_). Since LF variability of HR increases with sympathetic and decreases with parasympathetic activation and HF variability of HP increase with parasympathetic and decreases with sympathetic activation, the LF_HR_/HF_HP_ ratio may be a more sensitive measure of autonomic balance than LF_HP_/HF_HP_. Figure 7i–l shows the LF_HR_/HF_HP_ ratios for our experimental data. This ratio shows a decrease in response to taVNS (data from the three study days averaged, Figure 7l) and a decrease in response to OA‐D on the third study day (Figure 7k). These two findings are consistent with parasympathetic activation in response to taVNS and sympathetic withdrawal in response to OA‐D as demonstrated by the time‐domain and frequency‐domain analyses discussed above. Noteworthy, neither the LF/HF ratio calculated from HR time series, nor the LF/HF ratio calculated from RR‐interval time series detected the increase in parasympathetic tone in response to taVNS. Presumably, the decrease in sympathetic tone in response to OA‐D on Day 3 was only detected by LF/HF ratio calculated from HR or RR‐interval time series, because this intervention exclusively decreased sympathetic tone without significant effect on parasympathetic tone.

A limitation of our model is that we did not simulate changes in the threshold for Phase 0 depolarization which may occur in response to alterations in autonomic tone. We also did not consider changes in the amplitude of fluctuations in autonomic tone around the mean level of autonomic tone. It is possible that changes in the mean level of autonomic tone are accompanied by likewise changes in the amplitude of the fluctuations around the mean. If this were the case, variability measures calculated from HP and HR time series would likely show more similar trends. This may apply to gross autonomic maneuvers, such as head‐up‐tilt or stress tests, but may apply less to more subtle perturbations of autonomic function such as those in response to taVNA or OA‐D.

## 5 CONCLUSION

We developed a mechanistic framework for the interpretation of frequency‐domain HRV data that is based on a model simulation of sinus node automaticity. The framework may be applied to experimental LF and HF spectral powers calculated from heart period and heart rate time series according to Figure 4. Applying this framework to experimental data in humans suggest that taVNS results in an increase in cardiac parasympathetic nervous system activity and an associated reduction in heart rate by 4–5 bpm, while the osteopathic manipulative technique of OA‐D results in withdrawal of cardiac sympathetic tone without significant change in parasympathetic activity and a more modest reduction of heart rate in the order of 2–3 bpm. Based on these data, the effect of taVNS on cardiac parasympathetic tone lasts for at least 30 min, which was the duration of the recovery recording following the taVNS intervention. The response to OA‐D was more subtle and primarily seen during the OA‐D intervention on the third study day, although the increase in SDNN during the recovery recording (data from the three study days average) would suggest that the effect of OA‐D also lasted into the recovery recording. Finally, we provided a rationale for assessing autonomic balance by the LF to HF ratio (LF_HR_/HF_HP_) where LF is calculated from HR time series and HF is calculated from heart period time series.

## FUNDING INFORMATION

This study was supported by funding through the Office of Research and Sponsored Programs of Burrell College of Osteopathic Medicine, Las Cruces, NM and by the American Osteopathic Association (Grant No.: 19137759).

## CONFLICT OF INTEREST STATEMENT

The authors have no conflict of interest to declare.

## 6 ETHICS STATEMENT

This study was approved by the Institutional Review Board at Burrell College of Osteopathic Medicine (IRB# 0046_2019 and IRB# 0054_2019) and is registered with ClinicalTrials.gov (NCT04177264). All study participants provided written informed consent prior to participating in the studies.

## Data Availability

The data that support the findings of this study are available from the corresponding author, upon reasonable request and upon approval of data sharing by the Burrell College Institutional Review Board.
